# Verbal-Cue Rehabilitation Exercises Are Effective for the Mixed Callosal-Frontal Variant of Alien Hand Syndrome Following Stroke

**DOI:** 10.7759/cureus.68564

**Published:** 2024-09-03

**Authors:** Takumi Matsuyama, Koji Hayashi, Mamiko Sato, Asuka Suzuki, Yuka Nakaya, Toyoaki Miura, Yasutaka Kobayashi

**Affiliations:** 1 Rehabilitation Medicine, Fukui General Hospital, Fukui, JPN; 2 Health Science, Fukui Health Science University, Fukui, JPN

**Keywords:** callosal alien hand, cognitive-behavioral-therapy, cerebral infarction, verbal-cue rehabilitation exercise, alien hand syndrome (ahs)

## Abstract

Alien hand syndrome (AHS) is a rare condition in which a brain-injured patient develops involuntary movements, particularly in the upper limbs, along with difficulty in controlling them and a loss of ownership of the affected hand. AHS is traditionally classified into frontal, callosal, and occipital types. Recently, mixed types have also been reported. In this report, we describe a case of mixed callosal-frontal type AHS following stroke, in which symptoms improved with a verbal-cue rehabilitation exercise.

The patient was a 62-year-old woman diagnosed with cerebral infarction in the right frontal lobe and corpus callosum due to disruption of the right anterior cerebral artery. She exhibited left hemiplegia, left deep tendon hyperreflexia, and AHS in the left upper extremity. She received verbal-cue rehabilitation exercises for AHS in addition to regular rehabilitation therapy and medications. After treatment, AHS persisted but was remarkably attenuated.

## Introduction

Alien hand syndrome (AHS) is a rare symptom observed in patients with brain injuries, particularly in the upper limbs, where involuntary movements appear, making it difficult to keep the hand still, and the sense of ownership of the hand is lost [1]. In 1908, Goldstein first described this syndrome as "a type of apraxia accompanied by a sense of estrangement between the patient and the hand [2]." Since then, although it is a rare symptom, numerous case reports have accumulated, revealing its clinical features. AHS has been reported in association with underlying conditions such as stroke, brain tumors, post-corpus callosotomy, and degenerative diseases such as corticobasal degeneration [1,3]. Depending on the location of the lesion, it is classically classified into frontal type, callosal type, and occipital type [3]. While there is no precise definition of AHS, it is characterized by a sense of the hand (or foot) being alien, an inability to recognize ownership of the hand (or foot) when visual cues are removed, recognition of involuntary movements, and personification of the affected body part (feeling as if it is a separate entity) [4]. We report a case presenting with mixed callosal-frontal mixed type AHS associated with right anterior cerebral artery occlusion, where symptoms improved with verbal-cue rehabilitation exercises.

## Case presentation

**Figure 1:**
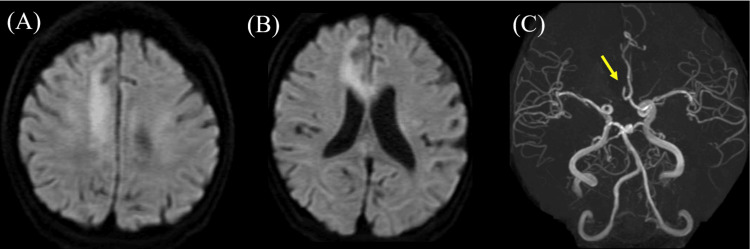
The result of brain MRI and MRA. (A, B) Diffusion-weighted brain MRI showing hyperintensity in the right frontal lobe convexity and corpus callosum. (C) MRA showing occlusion of the right anterior cerebral artery. MRI, magnetic resonance imaging; MRA, magnetic resonance angiography

A 62-year-old, right-handed Japanese woman developed disturbed consciousness after asthma attacks and was transported to the emergency room (ER). One day before this episode, she experienced numbness in her left lower limb following an asthma attack, but the symptoms disappeared after a short while. She had a history of diabetes, cerebral aneurysm, chronic heart failure, and neurogenic bladder. Her pre-morbid personality was impatient and optimistic. She had been receiving treatment for asthma since childhood, but the frequency of her asthma attacks had increased before hospitalization. Blood tests revealed elevated inflammatory markers (white blood cell: 14,500 /µL, C-reactive protein: 5.44 mg/dL), mild electrolyte abnormalities (sodium: 132 mEq/L, chlorine: 95 mEq/L), hypoalbuminemia (total protein: 5.5 g/dL, albumin: 2.6 g/dL), mild renal impairment (creatinine: 1.12 mg/dL), and liver dysfunction (alanine transaminase: 41 U/L, lactate dehydrogenase: 303 U/L, γ-glutamyltransferase: 119 U/L) (Table 1). Brain MRI revealed high signal intensity on diffusion-weighted imaging in the right anterior cerebral artery territory (Figures 1A, 1B), and MRA indicated occlusion of the right anterior cerebral artery (Figure 1C), leading to a diagnosis of cerebral infarction. Cervical ultrasound revealed intima-media thickness (IMT) of 1.0/1.0 mm in both common carotid arteries, indicating thickening, but no findings suggestive of significant stenosis within the visible range were observed.

**Table 1:** The result of blood tests on admission.

Inspection items	Result	Reference range
White blood cell count	14500 /μL	(3300-8600)
Red blood cell count	456×10⁴ /μL	(386-492×10⁴)
Hemoglobin	13.0 g/dL	(11.6-33.4)
Blood platelet	15.3×10⁴ /μL	(15.8-34.8)
Total protein	5.5 g/dL	(6.6-8.1)
Albumin	2.6 g/dL	(4.1-5.1)
Alkaline phosphatase	73 U/L	(38-113)
Aspartate aminotransferase	27 U/L	(13-30)
Alanine aminotransferase	41 U/L	(7-30)
Lactate dehydrogenase	303 U/L	(124-222)
Creatine kinase	44 U/L	(41-153)
γ-Glutamyltransferase	119 U/L	(13-64)
Total bilirubin	0.8 mg/dL	(0.4-1.2)
Direct bilirbin	0.1 mg/dL	(0-0.4)
Amylase	46 U/L	(44-132)
Choline esterase	274 U/L	(240-486)
Blood urea nitrogen	29 mg/dL	(8.0-20.0)
Uric acid	3.9 mg/dL	(3.7-7.0)
Creatinine	1.12 mg/dL	(0.46-0.79)
Sodium	132 mmol/L	(138-145)
Potassium	4.0 mmol/L	(3.6-4.8)
Chlorine	95 mmol/L	(101-108)
Glucose	130 mg/dL	(73-109)
C-reactive protein	5.44 mg/dL	(0.00-0.14)

It was determined that she was not a candidate for rt-PA or thrombectomy, so heparin was administered. After general management, including endotracheal intubation under sedation, she was managed in the intensive care unit (ICU). On the fourth day of hospitalization, her consciousness improved, but involuntary movements and hemiparalysis appeared in her left upper and lower limbs. Neurological examinations revealed that she was alert with an MMSE score of 28/30, displaying hyperactivity and restlessness. Higher brain function disorders included left-hand ideomotor apraxia, forced use of tools, initiation difficulties in the left upper and lower limbs, attention deficits, executive function disorders, disinhibition, interhemispheric transfer dysfunction, left-hand apraxia, agraphia, tactile naming disorder, and left hemispatial neglect. While looking at objects, she tilted her head, but there were no signs of ocular motor disturbances or diplopia, and no other cranial nerve abnormalities were observed. She had left hemiparesis (Brunnstrom stage: upper limb VI, fingers VI, and lower limb V), with mildly increased muscle tone in the left upper and lower limbs. Involuntary movements included writhing of the left hand against her will, which she was observed trying to restrain with her right hand. Deep tendon reflexes were mildly increased in the left limbs, and pathological reflexes included a grasp reflex in the left hand and a positive Rossolimo reflex on the left side. Although she could not concentrate well on sensory evaluation, and a detailed assessment was difficult, gross sensory deficits were not observed. No coordination disorders were detected. Her involuntary movement was evaluated as a mixed callosal-frontal variant of AHS because she developed difficulty in controlling movements of the left hand, as well as left-hand ideomotor apraxia, forced use of tools, attention deficits, executive function disorders, disinhibition, interhemispheric transfer dysfunction, left-hand apraxia, agraphia, astereognosis, and left hemispatial neglect. She was discharged from the ICU on the 10th day of hospitalization. From the 14th day, she was able to start oral intake, and clopidogrel 75 mg was initiated, but due to liver dysfunction, it was switched to aspirin 100 mg. On the 57th day of hospitalization, she was transferred to a rehabilitation ward. We planned the verbal-cue rehabilitation exercise according to a previous report (Qu et al.), which the guardian of the patient could effectively coordinate the movements of the hands of the patient through verbal cues, with the purpose of controlling AHS [5]. Specifically, whenever AHS symptoms appeared, rehabilitation therapists would inform the patient each time and use verbal cues to direct the patient's attention to it. Although left upper limb apraxia, ideomotor apraxia, forced grasping, and alien hand were observed, AHS improved with verbal-cue rehabilitation exercises. Difficulties initiating movements and inability to walk with the left lower limb were gradually addressed through activities of daily living (ADL) training and walking training. On the 81st day of hospitalization, she fell in her room, resulting in a femoral neck fracture, and underwent hip arthroplasty. Postoperatively, she made a good recovery and achieved independence in mobility through walking training. Although AHS persisted, it was within a range that did not interfere with daily life, and she was discharged home on the 201st day of hospitalization.

## Discussion

This case presented with corpus callosum-frontal mixed type AHS due to cerebral infarction, and verbal-cue rehabilitation exercise was effective. The lesion responsible for the cerebral infarction was in the right anterior cerebral artery, and the high signal intensity on diffusion-weighted imaging of the MRI study corresponded to its territory. This high signal region included the supplementary motor area (SMA) and corpus callosum. Abnormal blood test results, including elevated WBC and CRP levels on admission, may have contributed to the cerebral infarction. The patient exhibited difficulty in controlling the movements of the left hand, which is characteristic of AHS, as well as left-hand ideomotor apraxia, forced use of tools, attention deficits, executive function disorders, disinhibition, interhemispheric transfer dysfunction, left-hand apraxia, agraphia, astereognosis, and left hemispatial neglect.

AHS is classically categorized into frontal, callosal, and posterior types [6]. The frontal type appears when lesions are present in the medial frontal cortex of the dominant hemisphere, the SMA, the anterior cingulate gyrus, or the anterior part of the corpus callosum [3]. It is characterized by forced grasping and the forced use of tools, primarily with the dominant hand [3]. The SMA, located in the medial frontal lobe anterior to the primary motor cortex, plays a crucial role in the planning, initiation, and maintenance of movements [7]. When impaired, the grasp reflex, a symptom of frontal AHS, appears in the contralateral hand. The callosal type is characterized by ideomotor apraxia of the non-dominant hand due to lesions mainly in the corpus callosum [3]. The corpus callosum is responsible for the interhemispheric transfer of information, coordination, and inhibition pathways between the left and right hemispheres [8]. Different disconnection syndromes may occur depending on the anatomical part of the corpus callosum affected. Ideomotor apraxia and forced use of tools, symptoms of callosal, and frontal AHS, respectively, are associated with lesions in the anterior corpus callosum, including the rostrum, genu, and anterior body [8]. The posterior type appears when lesions are present in the thalamus, posterior cerebral artery territory, or parietal lobe, resulting in the loss of ownership and involuntary movements of the non-dominant hand (mainly the left hand), perceived as an "alien hand" [9].

Some reports of mixed types have emerged. The corpus callosum-frontal mixed type combines features of frontal and callosal types, while the corpus callosum-posterior mixed type combines features of callosal and posterior types [5,10]. These mixed types may occur when lesions are present in both the corpus callosum and the frontal or posterior lobes simultaneously. In this case, the patient exhibited grasp reflex and forced use of tools with the left hand, typical of the frontal type, as well as ideomotor apraxia and callosal disconnection syndrome, typical of the callosal type, leading to the diagnosis of corpus callosum-frontal mixed type. Although the frontal type of AHS is usually observed in the right hand (dominant hand), the reason it appeared in the left hand in this case requires further consideration [11]. We believe the primary condition was callosal type AHS, with additional features of frontal type (grasp reflex, forced use of tools) appearing in the left hand due to a lesion in the right frontal lobe. Tanaka et al. reported that when lesions extend to the anterior cingulate gyrus around the genu of the corpus callosum, abnormal behaviors such as forced use of tools with the contralateral hand are observed, regardless of which hemisphere is affected [7]. This finding applies to the forced use of tools observed in this case. Additionally, it is stated that the grasp reflex primarily results from lesions in the posterior part of the SMA on the contralateral side [7]. In this case, a lesion in the right SMA, contralateral to the left hand, was consistent with this explanation.

It has been reported that one of the rehabilitation therapies for AHS is cognitive behavioral therapy (CBT), which can help patients control the anger and frustration associated with their involuntary movements [12]. AHS is a condition in which the affected hand loses its sense of ownership [1]. Patients should first focus their attention on the affected hand, followed by gaining awareness and insight into it. Promoting self-regulation by intentionally using the affected hand is also important. We believe the goals of CBT for AHS are threefold: intentionally paying attention to the affected hand, recognizing the symptoms, and intentionally using the affected hand. We consider the verbal-cue rehabilitation exercise we employed to be a broad application of CBT. Verbal-cue rehabilitation exercise was first reported in a case of corpus callosum-frontal mixed type AHS due to cerebral infarction [5]. This approach involves verbally pointing out abnormal hand movements during AHS episodes, allowing the patient to consciously recognize and control these movements [5]. Repeated practice of this method helps patients become more aware of the affected hand and facilitates self-regulation of AHS episodes. This treatment incorporates the three components mentioned above, with verbal cues directing the patient’s attention to the affected hand, increasing awareness, and promoting intentional use of the hand. Therefore, verbal-cue rehabilitation exercise may aid recovery of AHS, through these mechanisms. We consider verbal-cue rehabilitation exercise to be one of the strategic options for treating AHS.

In our case, AHS was observed in the non-dominant left hand. Since the non-dominant hand is less frequently used intentionally in daily activities, it was more challenging to establish a cycle of use and feedback. Additionally, the patient had an optimistic yet impatient pre-morbid personality, exhibited poor insight, and struggled with disinhibition, attention deficits, and executive function disorders. Therefore, we initially anticipated that rehabilitation would be less effective and make recovery from AHS particularly challenging. However, through verbal-cue rehabilitation exercises, the patient was able to consciously control AHS within about a year, and the frequency of unconscious AHS episodes decreased, allowing her to perform daily activities. Moreover, verbal instructions and pointers enabled the patient to perform daily tasks safely and accurately, despite the difficulties posed by disinhibition, attention deficits, and executive function disorders. Thus, verbal-cue rehabilitation exercise was considered effective in addressing these frontal lobe symptoms.

The limitations of this study are primarily twofold. First, we were unable to establish a control group for verbal-cue rehabilitation exercises. AHS caused by cerebral infarction is rare, and the recovery process varies widely, from spontaneous improvement within a few months to chronic cases. Therefore, further case accumulation is needed to evaluate the effectiveness of verbal-cue rehabilitation exercises. Second, although this case involved mixed-type AHS, it remains unclear whether verbal-cue rehabilitation exercise is effective for other types of AHS. To generalize the effectiveness of verbal-cue rehabilitation exercises across all forms of AHS, a larger number of cases must be collected and analyzed.

## Conclusions

We presented a case of the mixed callosal-frontal variant of AHS that responded well to verbal-cue rehabilitation exercises. Verbal-cue rehabilitation exercise is a form of CBT. We believe that its effectiveness in treating AHS lies in the intentional focus on the affected hand, recognition of symptoms, and deliberate use of the affected hand. Further accumulation of cases is necessary to verify the effectiveness of verbal-cue rehabilitation exercises for AHS.
